# Association Between Eating Behaviors and Subjective Well-Being in Japanese Male Collegiate Handball Players

**DOI:** 10.3390/nu17193072

**Published:** 2025-09-26

**Authors:** Takaaki Nagasawa, Kumiko Minato

**Affiliations:** 1 Graduate School of Human Ecology, Wayo Women’s University, 2-3-1 Konodai, Ichikawa 272-8533, Chiba, Japan; 2 Department of Health and Nutrition, School of Human Ecology, Wayo Women’s University, 2-3-1 Konodai, Ichikawa 272-8533, Chiba, Japan

**Keywords:** handball, collegiate athletes, sports nutrition, eating behaviors, nutrient intake, subjective well-being, Hooper Score, breakfast, protein intake

## Abstract

**Background/Objectives**: Optimal well-being is critical for athletic performance, yet nutritional intake among athletes is frequently inadequate. Although subjective tools such as the Hooper Index are widely used to monitor athlete condition, their relationship with routine eating behaviors remains insufficiently explored. This study aimed to characterize the nutritional intake of Japanese male collegiate handball players and to identify eating behaviors associated with their subjective well-being, as measured by the Hooper Score. **Methods**: In this cross-sectional study, 64 male collegiate handball players completed a 3-day dietary record and a web-based questionnaire assessing eating habits, training load, and the Hooper Index (sleep, muscle soreness, stress, fatigue). Associations between dietary factors and the Hooper Score were examined using partial correlation and multiple regression analyses, adjusted for potential confounders. **Results**: Mean energy intake (30.1 ± 10.7 kcal/kg/day) and several micronutrient intakes were below recommended levels. Partial correlation analysis revealed that lower intakes of energy and multiple nutrients were significantly associated with poorer well-being (higher Hooper Scores) and more Subjective Health Complaints (SHC). Multiple regression analysis identified consistent dinner timing, greater protein intake (g/kg), more frequent consumption of nutrient-dense snacks, and less frequent consumption of unhealthy snacks as significant independent predictors of better Hooper Scores (*p* < 0.05). **Conclusions**: Suboptimal energy and nutrient intakes were common and associated with poorer subjective well-being. Specific eating behaviors, particularly meal regularity, snack quality, and adequate protein intake, emerged as independent predictors of the Hooper Score, offering practical indicators for nutritional assessment and athlete condition monitoring.

## 1. Introduction

In competitive sports, sustaining optimal performance requires a delicate balance between intensive training and adequate recovery [1]. Disruption of this equilibrium impairs adaptation to training stimuli and increases the risk of performance decline and injury [2]. Consequently, the continuous management of both physical and psychological conditions has become a central focus of athlete care.

In addition to objective physiological markers, subjective self-assessments that capture athletes’ own perceptions are widely recognized as practical and sensitive tools for monitoring subtle daily fluctuations in condition [3]. Among these, the Hooper Index has emerged as one of the most established instruments in sports science owing to its simplicity and high sensitivity in detecting responses to training load [4,5,6,7]. Variations in this index have been shown to correlate with post-match fatigue and changes in physiological parameters, reinforcing its utility as a reliable reflection of athletes’ condition [8]. By assessing four domains—sleep quality, muscle soreness, psychological stress, and fatigue—the Hooper Index provides comprehensive insights into both physical and mental states, thereby offering critical information for daily condition management, including overtraining risk and recovery needs [9].

Nutrition is a fundamental determinant of athletic condition, with particular relevance in sports characterized by high physiological demands. Handball exemplifies such a discipline, involving intermittent high-intensity activity (e.g., sprinting, jumping) interspersed with low-intensity aerobic movement (e.g., jogging) and frequent, forceful physical contact [10]. Players typically cover 3900–4700 m in a 60-min match [10], resulting in substantial energy expenditure and heightened requirements for recovery from muscle damage. These demands necessitate dietary patterns that ensure adequate nutrient intake aligned with the specific needs of the sport [11].

In addition to nutritional factors, the psychological state of athletes is a critical determinant of performance in handball. Evidence indicates that psychological interventions can enhance mental well-being and self-efficacy, thereby contributing to improved competitive outcomes [12,13]. Furthermore, attributes such as mental toughness and effective anxiety regulation have been identified as key differentiators between elite and sub-elite players [14]. Collectively, these findings emphasize that both physical and psychological domains are fundamental to success in handball, underscoring the importance of a holistic framework for athlete management.

Despite this, prior research has consistently demonstrated that handball players often fail to meet recommended nutritional standards. Reported deficiencies include insufficient carbohydrate intake, critical for glycogen replenishment, and inadequate protein intake, critical for muscle repair [15,16]. In addition, low consumption of vegetables and excessive reliance on nutritionally poor snack foods have been identified as common challenges [17,18]. Such dietary inadequacies contribute to low energy availability (LEA) and poor overall dietary quality, which may in turn exacerbate suboptimal physical and psychological condition [19]. The concept of Energy Availability (EA) provides a critical theoretical framework for understanding athlete health and performance. A state of LEA, arising from a mismatch between dietary energy intake and the energy expenditure required for training and daily life, is a primary cause of impaired physiological function and may manifest as the suboptimal conditions reflected by the Hooper Index. Collegiate athletes are particularly vulnerable given the unique pressures they face, including balancing academic responsibilities with training demands, independently managing their diets, and maintaining irregular daily schedules. These factors collectively place this population at increased risk of nutritional deficiencies and related adverse outcomes [20].

Nutritional and dietary challenges exert multifaceted influences on athletes’ subjective well-being. Regular meal consumption, for instance, has been closely linked to mental health outcomes; individuals who consistently consume three daily meals, including breakfast, demonstrate greater psychological stability and enhanced subjective well-being compared with those who engage in irregular eating patterns [21]. Similarly, dietary patterns rich in fruits and vegetables have been associated with higher levels of optimism and self-efficacy [22]. Furthermore, extensive evidence indicates that adherence to a Mediterranean diet and increased intake of omega-3 fatty acids, particularly from fish, are associated with a reduced risk of depression and improved mood states [23].

Dietary composition also exerts measurable effects on sleep and recovery. For example, consumption of a high-glycemic index carbohydrate meal before bedtime has been shown to facilitate sleep onset, while the intake of tryptophan-rich dairy products supports sleep stability [24,25]. With respect to muscle soreness, timely post-exercise protein ingestion promotes muscle repair, and the inclusion of anti-inflammatory food components, such as those present in berries and oily fish, may attenuate post-exercise inflammation and alleviate muscle pain [26,27].

Taken together, these findings suggest that eating behaviors—including meal regularity, overall dietary quality, and specific food selections—may influence both the physical and psychological domains that constitute the Hooper Index. Nonetheless, the majority of prior research involving athletes has emphasized the relationship between discrete nutrient intakes and objective outcomes such as performance metrics (e.g., time, distance) [28] or physiological markers of condition (e.g., blood biomarkers) [29]. Few investigations have systematically examined how an integrated set of eating behaviors, such as meal timing, meal skipping, and the qualitative nature of snack choices, relates to a comprehensive subjective indicator of well-being such as the Hooper Index. This gap is especially evident in handball, where research directly linking dietary patterns to psychological variables remains limited.

To address this gap, the present study aimed to characterize the nutritional intake and eating behaviors of Japanese male collegiate handball players and to identify practical behavioral indicators associated with subjective well-being, as measured by the Hooper Score. We hypothesized that easily measurable eating behaviors, such as meal regularity and snack quality, in combination with overall energy and nutrient intake, would serve as significant predictors of the Hooper Score.

## 2. Materials and Methods

### 2.1. Study Design

This study employed a cross-sectional design. Data were obtained through a web-based questionnaire and a 3-day dietary record to examine associations between eating behaviors, nutrient intake, and subjective well-being in collegiate handball players.

### 2.2. Participants

**Figure 1:**
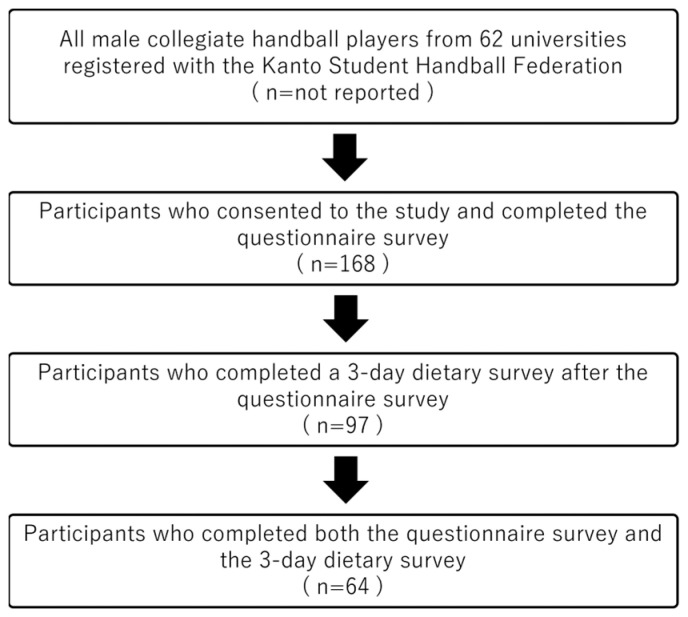
Flowchart of the participant selection process.

The participant selection process is illustrated in Figure 1.

Eligible participants were male collegiate handball players enrolled in universities registered with the Kanto Student Handball Federation in the greater Tokyo metropolitan area during the 2023 league season. Recruitment was initiated through formal invitations sent to all university handball clubs via the Federation office, supplemented by announcements at league competition venues. A total of 168 athletes consented to participate and completed a web-based questionnaire. All responses were complete, with no missing data. Of these, 97 players subsequently undertook a dietary survey. After exclusion of incomplete or missing dietary records, the final analytic cohort comprised 64 players who completed both the questionnaire and dietary components. Anthropometric data, including height and body weight, were self-reported through the questionnaire. The demographic characteristics and distribution by competitive level of the final 64 participants are presented in Table 1.

Participants represented a wide spectrum of competitive levels. Division 1 teams consisted of elite-level athletes, including several national team members, whereas lower-division teams primarily competed at a recreational level. Heterogeneity in competitive level was observed both across and within divisions. Variability also extended to institutional support structures: although some top-ranked teams employed athletic trainers, no participating team had access to a dedicated sports dietitian. The study protocol was reviewed and approved by the Research Ethics Committee for Human Participants at Wayo Women’s University (Approval No. 2329).

### 2.3. Dietary Survey

Dietary intake data were collected over 3 consecutive days, representing the participants’ typical schedule, including two training days and one rest day (in any order). A hybrid dietary record method was employed, combining estimated portion size documentation with photographic recording via a smartphone application. Participants were instructed to photograph all consumed food and beverages (excluding water) throughout the day, from waking to bedtime. For commercially prepared items and meals consumed outside the home, participants were additionally asked to photograph nutrition labels or menu descriptions that clearly displayed product names or ingredients. To complement the photographic data, participants recorded the time and location of consumption, dish and ingredient names, and estimated quantities. Additional details were required, such as whether dressing contained oil and the fat content of any milk consumed (e.g., low-fat or fat-free). Nutritional analyses were conducted by an experienced sports dietitian, who reviewed all dietary records and photographs to identify food items and estimate portion sizes. The energy and nutrient items assessed were selected based on those identified as potential challenges in athlete nutrition by the International Olympic Committee (IOC) [30], the American College of Sports Medicine (ACSM) [31], and the International Society of Sports Nutrition (ISSN) [32,33,34], with recommended values for athletes adopted from ACSM guidelines [31]. Energy and nutrient intake were calculated using Calorie Make (Version 1.0.10) and Eiyo Navi (Version 5.3.0) software (Toyo System Science Co., Ltd.), with nutrient composition data drawn from the Standard Tables of Food Composition in Japan, 2020 (8th Revised Edition). When submitted records were unclear or incomplete, participants were contacted directly for clarification.

### 2.4. Questionnaire Survey

The questionnaire was administered online through a secure web-based platform accessible via QR code. Items addressed dietary lifestyle, eating habits, training frequency and duration, weekly Rating of Perceived Exertion (wRPE), injury history, and Subjective Health Complaints (SHC). Questions on eating habits and SHC were adapted from instruments used in the authors’ previous studies [35,36] and selected items from the National Health and Nutrition Survey in Japan, with explanatory text provided for each item. For example, in questions concerning snack consumption, specific food examples were provided to facilitate accurate responses. To enhance clarity, “nutrient-dense snacks” were defined as foods beneficial for recovery and rich in carbohydrates, protein, vitamins, and minerals (e.g., rice balls, yogurt, fruit, milk), whereas “unhealthy snacks” were defined as foods high in fat and sugar but low in micronutrients (e.g., potato chips, chocolate, sugar-sweetened beverages). These definitions were made available to the participants within the questionnaire. The Subjective Health Complaints (SHC) checklist used in this study was developed by the authors, drawing on prior research in Japanese collegiate athletes [36,37]. It consists of 27 items reflecting the most frequently reported health complaints in this population, encompassing musculoskeletal pain, gastrointestinal issues, and general fatigue. For each item, participants indicated whether they had experienced the symptom (“Yes”) or not (“No”) within the past two weeks, and the total number of affirmative responses was used as the SHC score for analysis.

To ensure data completeness, the survey interface was designed to prevent progression until all items on a page were answered.

### 2.5. Hooper Index

The Hooper Index is a validated subjective tool designed to quantify perceived fatigue and general well-being in athletes [4]. It comprises four dimensions: sleep quality, muscle soreness, stress levels, and overall fatigue, each rated on a 7-point Likert scale. The cumulative Hooper Score ranges from 4 (reflecting optimal well-being) to 28 (reflecting poor well-being). This index is valued for its ability to detect meaningful variations in well-being that may not be identified by objective physiological markers [8]. Its high reliability, sensitivity to fatigue, and methodological simplicity have contributed to its widespread adoption in sports science research [5,6,7,38,39].

### 2.6. Weekly Rating of Perceived Exertion (wRPE)

To quantify internal training load, the wRPE was employed. Following the final training session of each week, participants were prompted to rate the overall intensity of their weekly training using the Borg CR-10 scale [40], where 0 represents complete rest and 10 denotes maximal effort. Daily RPE tracking was not feasible because athletes trained at various locations; however, this retrospective weekly approach was deemed appropriate based on prior validation studies demonstrating a strong correlation (r = 0.87) between weekly recalled RPE and the mean of daily RPE values [41].

### 2.7. Statistical Analysis

Normality of data distribution was assessed using the Kolmogorov–Smirnov and Shapiro–Wilk tests. Descriptive statistics are presented as mean ± standard deviation (SD). Differences between competitive divisions were assessed using one-way analysis of variance (ANOVA), followed, where significant main effects were observed, by Tukey’s HSD post hoc tests to identify pairwise group differences. Spearman’s rank correlation coefficients were calculated to examine associations between Hooper Score, SHC frequency, and energy/nutrient intakes. Partial correlation analyses were subsequently conducted, adjusting for potential confounders including competitive level, living environment, primary meal preparer, training frequency, duration, intensity, and receipt of dietary guidance. Multiple regression analysis was then performed with Hooper Score as the dependent variable.

All analyses were conducted using the Statistical Package for the Social Sciences (SPSS) for Windows, Version 28.0. Statistical significance was set at *p* < 0.05. Additionally, a post hoc power analysis was performed using G*Power 3.1 software [42] to evaluate the robustness of the regression model. The analysis, performed with an observed effect size of f^2^ = 0.976, an alpha level of 0.05, a total sample size of 64, and 6 predictors, demonstrated exceptionally high statistical power.

## 3. Results

Analysis of body composition by competitive division showed that players in Division 2 (*p* < 0.05) and Division 5 (*p* < 0.01) were significantly shorter than those in Division 1, with Division 5 players also being shorter than Division 2 players (*p* < 0.05). Body weight was likewise significantly lower in Division 2 (*p* < 0.05) and Division 5 (*p* < 0.01) compared with Division 1 (Table 2).

Analysis of dietary intake revealed that the mean total energy intake was 30.1 ± 10.7 kcal/kg body weight, a value below the threshold recommended for athletic populations. Although mean protein and carbohydrate intakes fell within recommended ranges, intakes of calcium, iron, and vitamins B1, B2, and D were consistently lower than advised levels. Comparisons across divisions revealed significant disparities: Division 2 players reported lower intakes of energy (kcal), protein (g), carbohydrates (g, g/kg), iron (mg), and vitamin B2 (mg) compared with Division 1. Division 5 players consumed significantly less energy (kcal), protein (g, g/kg), calcium (mg), vitamin B1 (mg), and vitamin B2 (mg) than Division 1, and calcium intake (mg) was also significantly lower in Division 5 compared with Division 2 (Table 3).

Analysis of training-related measures revealed that training frequency was significantly higher in Division 2 and Division 5 compared with Division 1 (*p* < 0.01). The Hooper score was also significantly higher in Division 5 compared with Division 1 (*p* < 0.05) (Table 4).

Partial correlation analyses, performed while adjusting for potential confounders, demonstrated significant negative correlations between both energy and multiple nutrient intakes and the Hooper Score, as well as with the number of SHC (Table 5).

Subsequent multiple regression analysis, with the Hooper Score as the dependent variable, identified several eating behaviors and nutrient intake factors as significant independent predictors (Table 6).

Specifically, a higher frequency of nutrient-dense snack consumption, lower frequency of unhealthy snack consumption, consistent dinner timing, and greater protein intake (g/kg body weight) were associated with better subjective well-being. Full details of the lifestyle and dietary survey results are pro-vided in Supplementary Table S1. Finally, a post hoc power analysis conducted using G*Power confirmed the robustness of these findings, demonstrating exceptionally high statistical power for the regression model (Power = 0.9999946).

## 4. Discussion

### 4.1. Association Between Overall Dietary Quality and Hooper Score (Subjective Well-Being)

The central finding of this study is that readily measurable, routine eating behaviors emerged as independent predictors of the Hooper Score, a validated index of subjective well-being. This observation is contextualized by the broader finding that participants’ energy and multiple nutrient intakes were consistently below recommended thresholds. Importantly, partial correlation analyses adjusted for potential confounders demonstrated that inadequate intake of both energy and a wide spectrum of nutrients was significantly associated not only with higher Hooper Scores, indicating poorer well-being, but also with an increased number of SHC. The observed associations were not confined to any single nutrient but encompassed a wide range, extending from macronutrients such as energy and protein to micronutrients essential for condition management, including iron and B vitamins. This pattern underscores that overall dietary quality, rather than isolated nutrient intake, is intrinsically linked to the maintenance of athletes’ physical and psychological condition. Consistent with this, a recent scoping review of athlete-focused studies concluded that overall dietary quality contributes to enhanced health, improved performance, and accelerated recovery [43]. The present study extends this evidence by highlighting a previously underexplored dimension: the association between comprehensive dietary quality and subjective well-being. Division level was also associated with well-being in our cohort, with athletes in lower divisions reporting poorer Hooper Scores. However, because this factor was statistically controlled for in the primary regression models, the observed associations between specific eating behaviors and the Hooper Score can be interpreted as independent of competitive level. These findings align with the EA framework introduced as the theoretical context for this study. Poor dietary quality, characterized by inadequate intakes of both macro- and micronutrients, likely contributes to a state of low energy availability, which is known to impair multiple physiological functions and would plausibly manifest as poorer subjective well-being, as reflected in the Hooper Score. These nutritional challenges observed in Japanese collegiate players appear to be consistent with findings from other regions. For instance, studies conducted among both adolescent [17] and elite [44] handball players have similarly reported inadequate energy and carbohydrate intakes, which may compromise athletes’ well-being and recovery. Taken together, these findings suggest that the association between suboptimal dietary patterns and poor subjective condition may represent a widespread issue in handball, regardless of geographical and cultural context.

### 4.2. Eating Behaviors Associated with Subjective Well-Being

Multiple regression analysis identified several eating behaviors, considered concrete indicators of overall dietary quality, as independent predictors of the Hooper Score. One such factor was meal regularity, where consistent dinner timing was associated with lower Hooper Scores, reflecting better subjective well-being. This finding highlights the critical role of meal timing in regulating circadian rhythms and maintaining physiological and psychological stability [45]. In contrast, irregular dinner timing may disrupt digestion during sleep and interfere with the secretion of sleep-related hormones, thereby compromising sleep quality—an essential domain of the Hooper Index [46]. Furthermore, late or inconsistent evening meals can suppress morning appetite and increase the likelihood of breakfast omission, compounding the adverse effects of irregular meal patterns on athletes’ overall condition.

Snack quality also emerged as a significant behavioral predictor, with a higher frequency of nutrient-dense snack consumption and reduced intake of unhealthy snacks both associated with lower Hooper Scores. Beyond their immediate nutritional contribution, snack choices can serve as behavioral indicators of athletes’ nutritional literacy and self-regulation [47]. Collegiate athletes, who often manage their diets independently amid fluctuating living conditions, time constraints, and financial pressures, are particularly vulnerable to reliance on readily available, nutrient-poor snacks [48]. Substituting such snacks for nutrient-dense options during the critical post-training recovery period may delay glycogen resynthesis and impair muscle repair, effects that can manifest as greater fatigue and muscle soreness in subsequent Hooper assessments [34].

Adequate protein intake (g/kg body weight) was likewise significantly associated with improved Hooper Scores. While this does not necessarily imply a direct causal effect of protein on subjective well-being, it suggests that protein intake may function as a proxy for broader dietary patterns that incorporate main dishes such as meat, fish, and eggs. These foods are rich sources of essential micronutrients, including iron and B vitamins, both of which were negatively correlated with Hooper Scores in this study [49]. Therefore, sufficient protein intake may enhance multiple dimensions of subjective well-being by facilitating muscle repair and reducing soreness [33], supporting energy metabolism [50], and preventing anemia-related fatigue [51]. This finding underscores the potential value of simple screening questions—such as whether the previous day’s meals included protein-rich main dishes—as practical indicators of dietary quality and subjective well-being, thereby reducing reliance on detailed nutrient calculations in applied settings.

### 4.3. Application of Findings in Sporting Contexts

Traditionally, the Hooper Score has been employed in sports science primarily to monitor athlete responses to training load and to detect associated changes in condition [5,6,7,32,33]. The present findings extend this utility by demonstrating that the Hooper Score is also influenced by daily eating behaviors. This observation highlights a new application of the Hooper Score: as a simple, practical screening tool for the early identification of nutritional and behavioral challenges. For example, when an athlete exhibits a deterioration in Hooper Score, practitioners should consider not only training-related factors but also modifiable eating behaviors identified in this study, such as dinner regularity and snack quality. Such an approach could facilitate the identification of underlying contributors to poor condition and inform timely, targeted interventions. Integrating the Hooper Score with key behavior indicators may therefore enhance the delivery of individualized nutritional support in fast-paced sporting environments.

### 4.4. Limitations and Future Directions

Several limitations must be acknowledged. First, the cross-sectional design of this study precludes causal inference regarding the relationship between eating behaviors and subjective well-being. It remains unclear whether favorable dietary practices improve well-being, or conversely, whether athletes with greater well-being are more likely to adopt healthier behaviors. Longitudinal and dietary intervention studies are required to clarify these causal pathways.

Second, the study population was restricted to male collegiate handball players within a single geographical region, which limits the generalizability of the findings. Associations between eating behaviors and the Hooper Score may differ across competitive levels, sport types, sexes, and age groups. Moreover, the participants may have been more health-conscious than the broader athlete population, potentially introducing selection bias. Future research should therefore include more diverse cohorts to enhance external validity.

Third, the study lacked detailed characterization of participants. Although differences in Hooper Scores were observed across competitive levels, granular information on sporting history (e.g., years of training experience, playing positions) and contextual factors (e.g., training environment, lifestyle, or access to professional support) was not collected. As a result, the mechanisms underlying inter-division differences could not be clarified and remain an important avenue for future research.

Fourth, anthropometric data, including height and body weight, were self-reported. While direct measurement would have been preferable, the logistical challenges of coordinating assessments for more than 60 athletes across multiple universities made this approach unfeasible. Reliance on self-reported data may have introduced reporting bias, and future studies should aim to incorporate standardized direct measurements wherever possible.

Finally, the study relied exclusively on subjective assessments and did not address the physiological mechanisms underlying the observed associations. Future investigations should therefore complement global subjective indicators such as the Hooper Index with more specific assessments of its individual components, as well as objective physiological markers (e.g., blood biomarkers). Such a multifaceted approach would help to elucidate how specific eating behaviors influence stress hormones, inflammatory pathways, and other biological mediators, and how these mechanisms translate into changes in subjective well-being.

## 5. Conclusions

This study examined the relationship between eating behaviors, nutrient intake, and subjective well-being, as measured by the Hooper Score in Japanese male collegiate handball players. The analysis demonstrated that many participants exhibited energy and multiple nutrient intakes below recommended levels, and that lower overall dietary quality was significantly associated with poorer Hooper Scores. Furthermore, multiple regression analysis identified specific eating behaviors—namely, consistent dinner timing, higher frequency of nutrient-dense snack consumption, lower frequency of unhealthy snack consumption, and adequate protein intake (g/kg body weight)—as independent predictors of subjective well-being.

These findings underscore that athletes’ perceived well-being is influenced not only by absolute nutrient intake but also by broader patterns of daily eating behaviors. In particular, the inclusion of protein-rich main dishes and the preference for nutrient-dense snacks emerged as practical proxy indicators of overall dietary quality. The behavioral markers identified in this study provide a novel and pragmatic approach for monitoring and assessing athlete condition in applied sports settings, where time and resources often preclude detailed nutritional analysis. Incorporating such indicators into routine practice may facilitate the early detection of athletes at risk of poor condition and support the delivery of more tailored and effective nutritional guidance.

## Figures and Tables

**Table 1:** Number of universities and participants by competitive division.

Division	Number of Teams (Universities)	Number of Participants
1st Division (high level)	4	24
2nd Division	8	14
3rd Division	1	5
4th Division	3	5
5th Division	5	10
6th Division	3	3
7th Division	2	3
Total	26	64

**Table 2:** Anthropometric characteristics of participants (*n* = 64).

		All Division (*n* = 64)	1st Division (*n* = 24)	2nd Division (*n* = 14)	3rd Division (*n* = 5)	4th Division (*n* = 5)	5th Division (*n* = 10)	6th Division (*n* = 3)	7th Division (*n* = 3)
Age		19.6 ± 1.2	19.4 ± 1.2	19.1 ± 1.1	21.2 ± 1.2	20.0 ± 0.6	19.3 ± 1.1	19.7 ± 0.7	20.0 ± 0.8
Height	(cm)	174.6 ± 6.5	178.7 ± 5.8	174.2 ± 4.9 *	173.4 ± 4.5	176.8 ± 4.2	169.6 ± 3.0 ^††,‡^	166.7 ± 4.8	166.6 ± 4.8
Weight	(kg)	72.3 ± 9.2	76.6 ± 7.9	69.9 ± 7.7 *	77.1 ± 6.4	71.6 ± 6.2	66.3 ± 9.7 ^††^	67.1 ± 2.9	61.7 ± 5.1
BMI	(kg/m^2^)	23.7 ± 2.3	24.0 ± 1.8	23.0 ± 1.7	25.6 ± 1.8	22.9 ± 1.6	23.0 ± 3.1	24.1 ± 2.9	22.2 ± 1.9

Mean ± SD: Divisions 3, 4, 6, and 7 were excluded from the analysis due to small sample sizes. Division 1 vs. Division 2, * *p* < 0.05; Division 1 vs. Division 5, ^††^ *p* < 0.01; Division 2 vs. Division 5, ^‡^ *p* < 0.05; SD: standard deviation. BMI, body mass index.

**Table 3:** Daily intake of energy and nutrients (*n* = 64).

		All Division (*n* = 64)	1st Division (*n* = 24)	2nd Division (*n* = 14)	3rd Division (*n* = 5)	4th Division (*n* = 5)	5th Division (*n* = 10)	6th Division (*n* = 3)	7th Division (*n* = 3)	Recommended Intake for Athletes *
Energy	(kcal)	2205 ± 820	2601 ± 873	1967 ± 633 *	2067 ± 743	2132 ± 560	1770 ± 788 ^†^	2073 ± 456	2422 ± 181	3000
	(kcal/kg BW)	30.1 ± 10.7	33.9 ± 11.2	28.0 ± 7.6	27.0 ± 10.7	30.0 ± 8.0	27.3 ± 11.5	27.5 ± 10.3	30.4 ± 12.1	
Protein	(g)	89.0 ± 33.8	104.2 ± 32.4	78.9 ± 28.2 *	100.1 ± 43.0	94.6 ± 27.8	60.9 ± 23.5 ^††^	84.6 ± 19.8	93.6 ± 9.9	
	(g/kg BW)	1.2 ± 0.4	1.4 ± 0.4	1.1 ± 0.3	1.3 ± 0.6	1.3 ± 0.3	1.0 ± 0.4 ^††^	1.3 ± 0.4	1.5 ± 0.1	1.2–2.0
	(%)	16.5 ± 3.5	17.2 ± 3.4	16.1 ± 2.6	18.9 ± 6.2	17.9 ± 0.6	14.0 ± 2.1	16.1 ± 3.2	15.5 ± 1.1	
Fat	(g)	75.2 ± 32.0	79.9 ± 32.4	72.9 ± 28.7	65.7 ± 21.9	68.0 ± 14.9	66.9 ± 43.0	89.2 ± 20.1	105.4 ± 12.7	
	(%)	30.6 ± 8.2	27.5 ± 7.6	32.4 ± 7.4	30.0 ± 7.9	28.5 ± 6.1	33.1 ± 8.9	37.0 ± 4.4	38.8 ± 1.9	20–35
Carbohydrate	(g)	313.1 ± 132.3	388.8 ± 149.9	267.4 ± 84.5 **	291.8 ± 111.4	302.9 ± 97.6	247.3 ± 100.7	252.6 ± 54.8	288.4 ± 13.8	
	(g/kg BW)	4.3 ± 1.7	5.1 ± 1.9	3.8 ± 1.0	3.8 ± 1.6	4.3 ± 1.4	3.8 ± 1.5	3.9 ± 1.1	4.7 ± 0.2	3.0–12.0
	(%)	53.5 ± 8.3	56.2 ± 8.1	52.9 ± 7.0	51.1 ± 5.6	54.4 ± 5.8	53.2 ± 10.1	46.3 ± 4.6	45.7 ± 2.2	50–65
Calcium	(mg)	339 ± 192	388 ± 186	293 ± 142	481 ± 299	307 ± 68	192 ± 59 ^††^	397 ± 215	351 ± 145	1300–1500
Iron	(mg)	7.3 ± 3.2	7.9 ± 2.8	5.8 ± 1.7 *	7.4 ± 5.3	9.7 ± 3.8	7.0 ± 3.3	7.2 ± 1.8	7.8 ± 1.2	15–18
Retinol Equivalent	(μg)	355 ± 182	369 ± 171	312 ± 132	283 ± 160	347 ± 119	335 ± 234	501 ± 181	502 ± 167	700–900
Vitamin B_1_	(mg)	1.30 ± 0.66	1.39 ± 0.52	1.13 ± 0.37	2.19 ± 1.23	1.56 ± 0.55	0.95 ± 0.47	1.02 ± 0.23	1.12 ± 0.21	1.50–3.00
	(mg/1000 kcal)	0.62 ± 0.30	0.56 ± 0.19	0.60 ± 0.23	1.10 ± 0.63	0.72 ± 0.14	0.56 ± 0.22	0.50 ± 0.10	0.47 ± 0.11	
Vitamin B_2_	(mg)	1.17 ± 0.68	1.32 ± 0.56	0.94 ± 0.25 **	2.15 ± 1.46	1.00 ± 0.22	0.74 ± 0.33	1.18 ± 0.38	1.36 ± 0.11	
	(mg/1000 kcal)	0.54 ± 0.29	0.52 ± 0.20	0.50 ± 0.11	1.05 ± 0.72	0.48 ± 0.06	0.42 ± 0.05	0.56 ± 0.12	0.57 ± 0.09	1.10
Vitamin C	(mg)	97 ± 151	110 ± 211	63 ± 36	164 ± 147	45 ± 16	103 ± 132	98 ± 53	135 ± 51	100–200
Vitamin D	(μg)	6.7 ± 5.8	7.0 ± 5.5	5.9 ± 5.0	11.8 ± 7.9	2.8 ± 1.0	4.9 ± 5.8	9.8 ± 4.2	11.0 ± 0.9	15–20
Fiber	(g)	19.5 ± 7.0	21.7 ± 7.2	17.5 ± 5.9	17.1 ± 4.7	18.3 ± 5.1	18.9 ± 8.7	19.5 ± 4.6	22.0 ± 5.2	

Mean ± SD: Divisions 3, 4, 6, and 7 were excluded from the analysis due to small sample sizes. Division 1 vs. Division 2, * *p* < 0.05, ** *p* < 0.01; Division 1 vs. Division 5, ^†^ *p* < 0.05, ^††^ *p* < 0.01; SD: standard deviation; Reference to the American College of Sports Medicine [31]; BW, body weight.

**Table 4:** Training frequency, duration, perceived exertion, and fatigue-related measures across divisions (*n* = 64).

Variable		All Division (*n* = 64)	1st Division (*n* = 24)	2nd Division (*n* = 14)	3rd Division (*n* = 5)	4th Division (*n* = 5)	5th Division (*n* = 10)	6th Division (*n* = 3)	7th Division (*n* = 3)
**Training Variables**									
Training frequency	(days per week)	3.3 ± 1.3	2.3 ± 0.7	3.6 ± 1.0 **	3.8 ± 1.2	4.0 ± 1.6	3.9 ± 0.4 ^††^	5.0 ± 0.6	5.0 ± 0.8
Training time	(hours)	3.5 ± 0.7	3.8 ± 0.6	3.2 ± 0.7 **	3.2 ± 0.4	3.2 ± 0.4	3.4 ± 0.8	3.2 ± 0.7	3.3 ± 0.5
**Perceived Exertion**									
Weekly RPE		5.0 ± 1.7	5.5 ± 1.8	4.9 ± 1.8	4.8 ± 1.9	6.0 ± 1.4	4.3 ± 1.3	4.2 ± 0.7	4.3 ± 0.5
**Fatigue-related Measures**									
Hooper Score		14.2 ± 3.3	13.4 ± 2.9	14.6 ± 4.0	13.2 ± 2.5	13.8 ± 3.4	15.9 ± 3.0 ^†^	14.7 ± 2.2	13.0 ± 1.6
Number of subjective health complaints		2.5 ± 2.2	1.8 ± 1.8	3.0 ± 2.9	2.4 ± 1.6	3.6 ± 2.2	2.6 ± 1.4	2.7 ± 2.1	1.7 ± 0.5

Mean ± SD: Divisions 3, 4, 6, and 7 were excluded from the analysis due to small sample sizes. Division 1 vs. Division 2, ** *p* < 0.01; Division 1 vs. Division 5, ^†^ *p* < 0.05, ^††^ *p* < 0.01; SD: standard deviation; BMI, body mass index.

**Table 5:** Partial correlation coefficients between dietary factors and fatigue indicators.

Variable		Hooper Score	Number of Subjective Health Complaints
**Fatigue and Symptoms**			
Number of subjective health complaints		0.531 *	
**Macronutrient Intake**			
Energy	(kcal)	−0.271 *	−0.369 **
	(kcal/kg BW)	−0.264 *	−0.377 **
Protein	(g)	−0.405 **	−0.403 **
	(g/kg BW)	−0.434 **	−0.450 **
Fat	(g)	−0.125	0.281 *
Carbohydrate	(g)	−0.268 *	−0.330 **
	(g/kg BW)	−0.268 *	−0.346 **
**Micronutrient Intake**			
Calcium	(mg)	−0.259 *	−0.252
Iron	(mg)	−0.334 **	−0.387 **
Retinol equivalent	(μg)	−0.107	−0.136
Vitamin B_1_	(mg)	−0.364 **	−0.132
	(mg/1000 kcal)	−0.017	−0.093
Vitamin B_2_	(mg)	−0.404 **	−0.242
	(mg/1000 kcal)	−0.025	0.088
Vitamin C	(mg)	−0.206	−0.195
Vitamin D	(μg)	−0.286 *	−0.269 *
Fiber	(g)	−0.289 *	−0.255 *

Competitive level, living environment, meal preparer, training frequency, training time, dietary guidance from a dietitian, and dietary guidance from non-dietitians were included as control variables as potential confounding factors. * *p* < 0.05, ** *p* < 0.01.

**Table 6:** Multiple regression and ANOVA results for Hooper score.

Predictor	B	Standard Error	β	*t*	*p*	95% CI_lower_	95% CI_upper_	Tolerance	VIF
(constant)	14.868	2.900		5.127	0.000	9.061	20.675		
nutrient-dense snacks	−1.536	0.757	−0.224	−2.028	0.047	−3.053	−0.019	0.730	1.369
Unhealthy snacks	4.041	1.326	0.297	3.048	0.003	1.386	6.695	0.937	1.067
Dinner time	−2.647	0.765	−0.340	−3.462	0.001	−4.178	−1.116	0.920	1.087
Protein (g/kg)	−4.942	1.437	−0.630	−3.439	0.001	−7.820	−2.065	0.265	3.779
Evaluation of my own diet	−1.337	0.718	−0.198	−1.863	0.068	−2.775	0.100	0.787	1.271
Energy (kcal/kg)	0.050	0.051	0.162	0.994	0.324	−0.051	0.152	0.334	2.991
**Model summary**									
**model**	***R***	***R* ^2^**	**Adjusted R^2^**	**SE of the estimate**	**df1**	**df2**	**R^2^ change**	***p***	
1	0.703	0.494	0.440	2.485	6	57	0.494	<0.001	
**ANOVA Source**									
**Source**	**Sum of squares**	**df**	**Mean square**	***F*-value**	***p*-value**				
Regression	343.109	6	57.185	9.260	<0.0001 ^a^				
Residual	352.000	57	6.175						
Total	695.109	63							

Footnotes: ^a^ Overall model *p* < 0.05 (two-tailed). Dependent variable: Hooper index.

## Data Availability

In accordance with the Personal Information Protection Law of Japan, individual participant data from this study cannot be made publicly available. However, data from this study will be made available to members of the scientific and medical community for non-commercial use upon publication, following a reasonable request via email to the corresponding author, Takaaki Nagasawa (email: nagasawa@wayo.ac.jp).

## Supplementary Materials

The following supporting information can be downloaded at: https://www.mdpi.com/article/10.3390/nu17193072/s1, Table S1: Dietary and lifestyle survey results among collegiate athletes.

## Abbreviations

The following abbreviations are used in this manuscript:

LEA	Low energy availability
SHC	Subjective Health Complaints
wRPE	weekly Rating of Perceived Exertion
